# The *FMR1* gene, infertility, and reproductive decision-making: a review

**DOI:** 10.3389/fgene.2014.00195

**Published:** 2014-07-07

**Authors:** Lisa M. Pastore, Joshua Johnson

**Affiliations:** ^1^Department of Obstetrics and Gynecology, School of Medicine, University of VirginiaCharlottesville, VA, USA; ^2^Department of Obstetrics, Gynecology and Reproductive Sciences, Yale UniversityNew Haven, CT, USA

**Keywords:** *FMR1*, female infertility, primary ovarian insufficiency, mouse models, diminished ovarian reserve, follicle stimulating hormone, anti-mullerian hormone, genetic counseling

## Abstract

The strongest association between *FMR1* and the ovary in humans is the increased risk of premature ovarian failure (POF) in women who carry the premutation level of CGG repeats (55–199 CGGs). Research on the *FMR1* gene has extended to other endpoints of relevance in the OB/GYN setting for women, including infertility and ovarian hormones. After reviewing the nomenclature changes that have occurred in recent years, this article reviews the evidence linking the length of the *FMR1* repeat length to fertility and ovarian hormones (follicle stimulating hormone and anti-mullerian hormone as the primary methods to assess ovarian reserve in clinical settings). The literature is inconsistent on the association between the *FMR1* trinucleotide repeat length and infertility. Elevated levels of follicle stimulating hormone have been found in women who carry the premutation; however the literature on the relationship between anti-mullerian hormone and the CGG repeat length are too disparate in design to make a summary statement. This article considers the implications of two transgenic mouse models (FXPM 130R and YAC90R) for theories on pathogenesis related to ovarian endpoints. Given the current screening/testing recommendations for reproductive age females and the variability of screening protocols in clinics, future research is recommended on pretest and posttest genetic counseling needs. Future research is also needed on ovarian health measurements across a range of CGG repeat lengths in order to interpret *FMR1* test results in reproductive age women; the inconsistencies in the literature make it quite challenging to advise women on their risks related to *FMR1* repeat length.

## *FMR1* gene overview

The name “fragile X” refers to a cytogenetic abnormality on the long arm of the X chromosome. The genetic focus of fragile X research is the 5′-untranslated region of the fragile X mental retardation 1 (*FMR1*) gene, located at Xq27.3.

The reference range for the *FMR1* trinucleotide repeat length has four categories. The trinucleotide repeat reference intervals are based on studies designed to establish the diagnosis of Fragile X Syndrome (FXS) and the associated risk of expansion to a full mutation that occurs during transmission from a premutation carrier mother to her offspring. FXS is the most common heritable form of intellectual and developmental disabilities and autism. The categories in order of increasing trinucleotide length are normal, intermediate, premutation, and full mutation. The CGG repeat size corresponding to each diagnostic category generally varies by ±5 repeats in the literature due to changing definitions over time and differences in individual report categories. The current categories are defined as follows:

- The American College of Obstetrics and Gynecology Genetics Committee Report, the American College of Medical Genetics (ACMG) Quality Assurance Committee and the ACMG Technical Standards Report all explicitly state that an *FMR1* CGG repeat length less than 45 is not associated with an abnormal phenotype (Kronquist et al., 2008; ACOG Committee of Genetics, 2010; Monaghan et al., 2013). Thus, <45 CGG repeats is considered normal.
- Repeats in the range of 45–54 are termed intermediate or “gray zone” or inconclusive (Maddalena et al., 2001; Kronquist et al., 2008; Monaghan et al., 2013).
- Repeats in the range of 55–199 are termed “premutation” (Maddalena et al., 2001; Kronquist et al., 2008).
- At least 200 CGG repeats is a “full mutation” and results in FXS in the majority of boys and in some girls with this genetic mutation. A gene with this CGG repeat level is typically hypermethylated, resulting in transcriptional silencing such that no or only low levels of protein (FMRP) are produced (Oostra and Willemsen, 2003).

In terms of clinical phenotypes that have been associated with this gene, as stated above, full mutations can cause FXS. As research demonstrated that many mothers of FXS boys were carriers of a premutation-size allele, the importance of the premutation category and potentially the intermediates became recognized by clinical authorities. This is reflected in statements by ACMG committee members: “the clinical significance of intermediate and low premutation size alleles… is the extent to which they may be prone to instability, particularly expansion, in future generations” (Kronquist et al., 2008). For a review of the mental health impact of the *FMR1* gene, both in terms of the affected individual and their family, the reader is referred to Seltzer et al (Seltzer et al., 2009).

The *FMR1* gene has been associated with alterations of some reproductive milestones. Most notable is the evidence that premutations are associated with premature ovarian failure (POF). The *FMR1* premutation is present in about 11% of familial POF cases and about 3% of sporadic POF cases (Marozzi et al., 2000; Murray et al., 2000; Bussani et al., 2004), although a recent report from the United Kingdom suggests that only 2% of POF cases have a premutation (Murray et al., 2014). The odds of being a premutation carrier if the woman is postmenopausal before age 40 was recently estimated to be more than fivefold from that UK report (Murray et al., 2014).

Following the confirmation of the association between the *FMR1* premutation and POF, publications appeared on the relationship between ovarian hormones, infertility and this gene. Not surprisingly, the volume of research on hormones and fertility is far smaller than the volume on POF, and this body of research is the focus of this publication. Before we summarize the literature, comments on nomenclature are warranted.

## What is primary ovarian insufficiency (POI)? terminology and confusion

POF and diminished ovarian reserve (DOR) are clinical diagnoses made by reproductive endocrinologists (Fritz and Speroff, 2011a,b); POF is also a recognized clinical diagnosis made by endocrinologists in general (Jameson and DeGroot, 2010). POF is diagnosed by three characteristics: postmenopausal levels of follicle stimulating hormone (FSH) (>40 IU/L), 4 or more months of secondary amenorrhea, and age <40 years (Coulam et al., 1986). DOR is diagnosed by elevated FSH levels (>10 IU/L in cycle days 2–4, or a failed clomiphene citrate challenge test) and regular periods (Fritz and Speroff, 2011a), although some clinics use anti-mullerian hormone and/or antral follicle count in their assessment of ovarian reserve. DOR is a normal physiologic process when it occurs in the mid to late forties, and is pathological at younger ages.

Around 2007–2008, the term primary ovarian insufficiency (POI) was suggested to represent a continuum of dysfunction related to early aging of the ovaries. Controversy exists as to its precise definition, however. Some notable clinicians and researchers (De Vos et al., 2010) have defined POI the same as POF. The terminology of POI is considered to better represent this condition, considering that there is a progression of ovarian dysfunction that precedes the premature amenorrhea (Welt, 2008), as well as the fact that women with this condition sometimes spontaneously have follicular development and/or returned menses and/or conceive after the diagnosis is made (Rebar and Connolly, 1990; Nelson et al., 1994).

Other clinicians/researchers have suggested that POI represents a continuum of ovarian conditions that encompass an “occult” clinical state (reduced fecundity but normal FSH levels and regular menses), “biochemical” state (reduced fecundity, elevated FSH and regular periods), and an “overt” state (approximately corresponding to POF though perhaps with irregular menses) (Welt, 2008).

It is interesting to note that neither ICD-9 nor ICD-10 medical coding systems use the POF or POI terminology. ICD-9 uses the term “premature menopause” (code 256.31). For ICD-10, there are 2 corresponding codes: E28.310 “Symptomatic premature menopause” and E28.319 “Asymptomatic premature menopause.” The description of E28.310 is “Symptoms such as flushing, sleeplessness, headache, lack of concentration, associated with premature menopause.” The description of E28.319 corresponds to the current ICD-9 code of 256.31. The billing code for DOR in ICD-9 is 256.8 (“Other ovarian dysfunction”), and this remains constant in ICD-10.

In terms of Index Medicus, “primary ovarian insufficiency” is now a MESH term under the category of Ovarian Disease, as is also true for “Premature Menopause.” There is not a MESH term for DOR.

Clearly there are differences in terminology, which are beyond the scope of this paper to resolve. This terminology is reviewed here in a manner heretofore not discussed in the literature, in order to address confusion among readers from disparate backgrounds. For this publication, we will use the clinical diagnosis terms of POF and DOR for the research on humans.

## Methodologic issues in the human *FMR1* studies

### Unit of analysis

Two of the studies reviewed in Table 1 use alleles rather than women as the unit of analysis. It is important for readers to note whether a given report is estimating the allele frequency or the carrier frequency in the interpretation of the results and when comparing results across studies. For example, an analysis that uses both alleles has the effect of inflating the sample size by two, because all women contribute two chromosomes and, thus, 2 *FMR1* alleles. Since the majority of alleles are in the normal range, this approach will underestimate the frequency of intermediate and premutation repeat lengths among women. It is not possible to make a general statement on the impact on the statistical significance of the findings that use both alleles rather than one allele. We have noted via italics in Table 1 when a study used alleles rather than the woman as the unit of analysis.

**Table 1:** **Literature summary on *FMR1* CGG repeat length among women diagnosed with fertility delays due to ovarian reserve issues, sorted by year**.

**First author year; country**	**Study sample description**	**Key results**		
Streuli et al., 2009; Switzerland	Source: infertility practices	*Note: Analysis compares allele frequencies, not carrier frequencies.*		
	Sample: 27 cases with low ovarian reserve (high FSH or low AMH or poor response to stimulation) vs. 32 controls (referred for genetic testing unrelated to fertility or mental impairment)	Mean CGG length is higher in 27 cases (54 alleles, 33 CGG) than controls (64 alleles, 28 CGG). Similar results for biallelic mean.		
	*IM* = 41–60 CGG		Cases	Control
		NC	47	63
		IM	4 (7.4%)	1 (1.6%)
		PM	3 (5.6%)	0
		Associations with IM and PM are not significant.		
		If dichotomize at >40 repeats, difference is significant;		
		*OR* = 9.4 (95% CI 1.1–81.2).		
		OR for 35–60 repeats is 5.4 (95% CI 1.1–25.6).		
Gleicher et al., 2009a; USA	Source: infertility clinic	The risk for low ovarian reserve (AMH ≤0.8 ng/mL) increased by 40% for every +5 higher repeats above 30 CGGs on the higher allele (logistic regression adjusted for age, *p* = 0.02).		
	Sample: 316 fertility clinic patients, stratified by CGG repeat.			
	Analyses independently considered lower and higher alleles.			
Karimov et al., 2011; USA	Source: infertility clinic	Carrier frequency of PM and IM more common in women with low ovarian reserve than controls.		
	Sample: 535 cases with low ovarian reserve (elevated FSH, elevated day 2–4 estradiol, poor response to stimulation)		Cases	Controls
	vs. 521 controls (infertility unrelated to ovarian reserve, and oocyte donors).	PM	7 (1.3%)	1 (0.19%)
			*p* = 0.036 (vs. NC)	
	*IM* = 45–54 CGG	IM	17 (3.2%)	7 (1.3%)
			*p* = 0.046 (vs. NC)		
Pastore et al., 2012; USA	Source: infertility clinics	Carrier frequencies of 35–39 CGG and 40–44 CGG among cases with DOR was significantly greater than literature comparison groups.		
	Sample: 62 cases with DOR (elevated FSH or <6 antral follicles), age ≤42, regular menses, excluded family hx of FXS or PM.			
	Compared with 564 control women from literature [32 controls from Streuli et al. (2009), 162 from Bretherick et al. (2005), 370 from Otsuka et al. (2010), and 200 from Bodega et al. (2006)].
			35–44 CGGs	
		DOR	14.5%	
		Prior research	3.9% (*p* = 0.0003)	
Barasoain et al., 2013; Spain	Source: OB/GYN clinic patients	*Note: Analysis compares allele frequencies, not carrier frequencies.*		
	Sample: 22 cases with low ovarian reserve (irregular cycles, elevated FSH)	Low ovarian reserve case group more likely to have ≥35		
	vs. 7 women with POF	CGGs than controls (*p* < 0.05).		
	vs. 32 controls with no infertility and natural age at menopause			
	*IM* = 35–54 CGG		IM 35–54	PM
		Cases	4/44 (9%)	2/44 (5%)
		POF	3/14 (21%)	0/14
		Controls	2/64 (3%)	0/64
De Geyter et al., 2013; Switzerland	Source: Infertility patients (cases) and women with recent deliveries (controls)	IM and PM carrier frequencies not significantly different between groups.		
	Sample: 372 cases with infertility (excluding male or tubal factor infertility)		Infertile	Fertile
		NC	303	170
	vs. 199 fertile women (recent birth and natural conception within 3 months)	IM 35–44	55 (14.8%)	24 (12.1%)
		IM 45–54	9 (2.4%)	4 (2.0%)
	IM (*2 definitions analyzed*)	PM	5 (1.3%)	1 (0.5%)

PM, premutation; PMC, individual who carries the premutation; NC, non-carrier; POF, premature ovarian failure; DOR, diminished ovarian reserve; OR, odds ratio; FSH, follicle stimulating hormone; AMH, anti-mullerian hormone; hx, history; dx, diagnosis; IM, individual with an intermediate repeat length.

### Sample selection

Much of the research on the premutation and ovarian function derives from women with a family history of FXS. This is a practical population from which to draw a sample given the enrichment of premutation carriers in a family with a relative who has a full mutation. However, the biology underlying familial cases vs. sporadic cases of a given phenotype may very well be different. [For example, the likelihood of a premutation expanding to the full mutation in a single generation is higher in families with a family history of FXS than in families without a FXS relative (Nolin et al., 2011)] Thus, the findings of each type of study sample should be interpreted in the context of the population from which the study sample was drawn, noting that findings from FXS family studies may or may not extend to women without that family history.

### Range of repeat length under investigation

The research on *FMR1* has also changed over time in the repeat length of interest. The original research on FXS would, of course, be focused on full mutations with >200 CGG repeat lengths. When it was observed that there was a noticeable proportion of the premutation carrier mothers experiencing POF, the focus of some research shifted to the premutation range. With the advent of consistent and affordable laboratory testing for the exact repeat lengths, the possibility of analyzing the entire repeat range became the norm. The variation in the CGG repeat length of interest between reports can cause confusion among clinicians and patients regarding which genotype is or is not associated with a given phenotype. Additionally, inconsistencies in the literature may be due to differences in the repeat length being investigated between different publications, so readers should be cognizant of this issue.

## Literature on infertility and the *FMR1* gene

The literature on the prevalence of *FMR1* premutations in women with DOR is still at a very early stage (Table 1). Among *n* = 65 US cases of DOR, 14% had a high normal (35–44 repeats) *FMR1* CGG repeat length (Pastore et al., 2012). Their population had regular menses and no family history of FXS, and neither anti-Mullerian hormone (AMH) nor poor gonadotropin response was used to define cases. The proportion of high normal carriers was quite similar to a report from France (Streuli et al., 2009), where 17% of *n* = 27 women had 35–44 CGG repeats. In the French publication, cases were defined as having regular or irregular cycles (63/37%, respectively), FSH > 10 IU/L and/or AMH < 7 pmol/L and/or poor response to controlled ovarian hyperstimulation, and no family history of FXS. Among 22 women with DOR from Spain, 13.6% had ≥35 CGG repeats v 3.1% of the normal controls (*p* < 0.05) (Barasoain et al., 2013). A US study analyzed 535 women with a liberal definition of DOR (because cases could have skipped only 3 periods) compared to 521 controls (infertility from other causes or oocyte donors) (Karimov et al., 2011). Premutations and intermediate alleles were more commonly found in the cases than the controls (*p* = 0.036 and *p* = 0.046, respectively). A different US study (Gleicher et al., 2009a) reported an increased likelihood of DOR among women seen in their large fertility clinic with <28 CGG repeats as well as ≥34 repeats, with increasing risk the further there is an allele from a modal repeat length of 29–30 CGG's (*p* < 0.0002 among a cohort of 316 consecutive fertility patients). Among 372 infertile women from all causes (excluding those with complete tubal infertility), a recent report found no difference in the repeat distribution compared with 199 fertile controls in Switzerland (De Geyter et al., 2013). Clearly, it is still an outstanding question whether or not the *FMR1* gene is associated with low ovarian reserve and infertility, and if it is, which repeat length confers the greatest risk.

## Clinical markers for ovarian reserve (FSH and AMH) and *FMR1*

FSH, a gonadotropin under negative feedback of two ovarian hormones, inhibin B and estradiol (Burger, 1994, 2000), indirectly reflects the quantity of the antral follicles. It may also provide an indirect measure of the size or quality of the underlying follicle pool (Goldenberg et al., 1973; Klein et al., 2000). As ovarian function fails and the final menstrual period nears, the ovaries produce less inhibin and estradiol, allowing levels of unopposed FSH to rise (Burger et al., 2000). Among cycling women, early follicular phase FSH begins to increase (indicating diminished ovarian activity) beginning in the late 30 s to early 40 s (Kline et al., 2005; van Rooij et al., 2005).

In a small but informative US study (Welt et al., 2004), daily blood samples were examined for 1 month in 11 cycling premutation carriers aged 24–41 years. Their comparison group was 22 age-matched cycling women without a suggestive family history of FXS; *FMR1* testing was not conducted on the controls. Compared with the controls, women with a premutation allele had elevated FSH in both the follicular and luteal phases. Other reports with single FSH measures have also reported that a premutation allele is associated with elevated FSH levels (Murray et al., 1999; Hundscheid et al., 2001; Sullivan et al., 2005). No differences in FSH (single measurement) was found between women with a full mutation and those with <60 CGG repeats (Murray et al., 1999). A recent report with 372 infertile women from all causes, of whom 9 had an intermediate allele and 5 had a premutation, reported no association between FSH and the CGG repeat level (De Geyter et al., 2013). Among fertile women in the US (Kline et al., 2014), there was no association between intermediate alleles (defined as 35–54 CGG) and hormones (AMH and FSH) in fertile women; These observations held true in their total sample of 583 pregnancies, as well as when restricted to 325 pregnancies that ended in a live birth.

AMH, also known as Mullerian-inhibiting substance, is a dimeric glycoprotein. It is produced by Sertoli cells of the testis in males and by ovarian granulosa cells in females. AMH is increasingly used by clinicians to measure ovarian reserve in pre-menopausal women and the volume of publications using AMH measurements has significantly increased in the past 5 years. As reviewed by Nelson and La Marca (Nelson and La Marca, 2011), AMH has several unique characteristics, including that it appears that circulating AMH in females is produced solely by the ovarian granulosa cells from primary to small antral follicles (≤4–6 mm) (Weenen et al., 2004; Visser and Themmen, 2005). In a sample of 42 ovaries obtained by oophorectomy, the age-adjusted correlation between serum AMH and the natural log of the number of primordial follicles was 0.48, supporting the view that AMH reflects, in part, the size of the oocyte pool (Hansen et al., 2011).

Given that *FMR1* is associated with early ovarian aging and AMH is increasingly used as a measure of ovarian reserve, there are now some publications that have investigated AMH levels by *FMR1* CGG repeat. The scant literature on the topic of AMH and *FMR1* (six publications that adjust for the woman's age) is inconsistent. Three papers reported an inverse association between AMH and *FMR1* repeat after controlling for age. Among 158 consecutive cycling infertility patients (none of whom carried the premutation) under age 40 at a single center in the US (Gleicher et al., 2009b), AMH was lower in women with 35–50 CGG repeats (*n* = 35) than in women with <35 repeats (*n* = 122, *p* = 0.025). Using a population that combined women from the general female population and women with a family history of FXS in the US, AMH was lower in women with ≥70 CGG repeats compared to those with <70 repeats aged 31–40 years (*p* = 0.015); No association was found among women over age 40 or ≤30 (*p* > 0.08) (Rohr et al., 2008), and this may be due to the exclusion of women who were using hormone treatment. In a study that combined FXS family data from The Netherlands and the US, premutation carriers were found to have lower AMH levels than non-carriers at all ages (multi-level modeling, *p* < 0.0001) (Spath et al., 2011); As expected, AMH declined with increasing age among both premutation carriers and non-carriers. One paper reported a positive association between AMH level and *FMR1* repeat length after controlling for age: among 197 Korean women “at high risk” of diminished ovarian function, where the highest CGG repeat was 51 (Choe et al., 2013), a positive correlation was found between AMH and the CGG repeat length (*p* = 0.008). Two recent papers reported no association: a recent report with 372 infertile women from all causes reported no association between AMH and the CGG repeat level (De Geyter et al., 2013), and the sample included some women with premutation and intermediate length alleles. Among 532 fertile women in the US (Kline et al., 2014), there was no association between intermediate repeat lengths (defined as 35–54 CGGs) and AMH. Recent modeling by the authors (Pastore et al., 2014a), with data from 79 women with DOR in the US, found that a linear model of log (AMH), corresponding to an exponential decline of AMH with increasing age, was significantly different, and had a steeper slope, for women with ≥35 CGG repeats than women with <35 repeats (*p* = 0.035).

These FSH and AMH observations, while intriguing, have limitations: studies are few; sample sizes are small; not all account for the age of the woman; most use cross-sectional data; and most studies exclude women who reached menopause, thereby selecting for women who do not show the most severe effects of the premutation. Differences in the results may be due to the populations studied (FXS families, fertile women, or infertile women).

## Animal models relevant to these human phenotypes

Developing appropriate animal and cell models of ovarian effects from the *FMR1* gene are vitally important, as research in humans is limited by the availability of specimens and participants, as well as the practicality of harvesting tissues like the ovary for analysis. Vertebrate (mouse and rat) and invertebrate (fruit fly; *Drosophila melanogaster*) animal studies related to the *FMR1* premutation and ovarian function exist and have been instrumental in advancing our understanding of the disease phenotype, as recently reviewed (Sherman et al., in review). Nonhuman primates (NHP) offer a clinically relevant model system in which to explore the molecular mechanisms of the premutation on ovarian function. The Washington National Primate Research Center is currently generating a nonhuman primate transgenic model of ovarian aging under the direction of Dr Eliza Curnow. Below, we summarize the most mature animal model data from two recent publications assessing the potential ovarian phenotypes of two distinct mouse models engineered to have *FMR1* PM repeats.

The female reproductive phenotypes of two transgenic mouse lines harboring PM repeats have been characterized. Usdin and colleagues inserted 130 CGG:CGG repeat lengths in the endogenous *FMR1* gene (referred to here as FXPM 130R; 130R), and Sherman and colleagues characterized a mouse line carrying a human premutation allele (derived from a yeast artificial chromosome) of 90 repeats (referred to as mouse line TG206). The female reproductive phenotypes that result from the two alleles are summarized in Table 2.

**Table 2:** **Mouse Models related to Ovarian Dysfunction and *FMR1***.

**Mutant phenotype**	**130R (Hoffman et al., 2012)**	**TG206/YAC90R (Lu et al., 2012)**		
Alteration(s) in follicle number	Decreased numbers of primordial follicles in 7–9 month-old mice	Decreased numbers of growing follicles in 25 day-old and 9 week-old mice		
Alteration(s) in fecundity	Not evaluated	Increased age at first litter, decreased number of pups per litter		
Alteration(s) in hormone production	Not evaluated	**Hormone**	**8–10 weeks**	**15–22 weeks**
		Estradiol	↑*	**
		FSH	↑*	↑*
		LH	↓ n.s.	↓ n.s.
		^*^P < 0.05, ^**^not evaluated		
Follicle death	Increased histomorphometric evidence of follicle atresia in PM ovaries; increased ubiquitylated proteins in PM oocytes.	Increased numbers of TUNEL-positive apoptotic cells in PM ovaries		
Alterations in follicle growth	Granulosa cell number is decreased in growing follicles. Cumulus granulosa cell number is also decreased in mature follicles.	mTOR pathway is downregulated in whole ovaries (McLaughlin et al., 2011; Yu et al., 2011)		
Alterations in gene expression	Reduced expression of FMRP in PM granulosa cells. Redistribution of FMRP to oocyte nucleus in PM (FMRP is cytoplasmic in wild-type oocytes). Reduced CX36 expression.	Decreased expression of LH-induced ovulation-related genes in PM ovaries versus wild-type controls.		
Other abnormalities	Presence of ovarian cysts; zona pellucida abnormalities.	Reduced whole body weight in PM animals versus wild-type controls.		

Fertility and fecundity were the primary measures of the reproductive status of the mouse lines, accompanied by analyses of follicle number, survival, and ovarian gene expression versus same-strain wild-type controls. In both mouse lines, fertility and fecundity were largely normal, and only YAC90R females demonstrated a slight but significant increase in the age at first litter, and a decreased number of pups per litter (Lu et al., 2012). While mouse age at first litter and slightly decreased fecundity in terms of litter size do not have direct clinical correlates, these findings hint at ovarian dysfunction in mice. To test whether the premutation negatively affects oocyte survival (and might thereby accelerate the demise of ovarian function), follicles were counted in both mouse strains.

While identical follicle counts were not performed on the two mouse strains, in each case some differences were seen compared to wild-type controls. Ovaries of the 130R mice were evaluated at several postnatal time points, including mice 10–12 months of age. Interestingly, only mice between 7 and 9 months of age showed any difference in follicle numbers, a significant decrease in primordial follicles compared to same-strain wild-type controls. A decrease in primordial follicles was not seen, however, in the mutants between 10 and 12 months. The follicle counts performed on the YAC90R mice revealed that there were fewer growing follicles in the ovaries of 25 day old and 9 week old mutants versus controls. This corresponded to their delivering fewer pups per litter than wild-type animals.

Despite the absence of an overt acceleration in follicle loss in the YAC90R and 130R mice, there were some differences in follicle development and granulosa cell survival that are suggestive of dysfunction. Increased histomorphometric evidence of follicle atresia (e.g., granulosa cell pyknosis) was seen in 130R PM ovaries, which was matched by increased numbers of TUNEL-positive apoptotic cells seen in the follicles of YAC90 PM ovaries. Hoffman et al. performed a careful analysis of the number of granulosa cells within individual 130R follicles and found that there were fewer granulosa cells in mutant follicles (Hoffman et al., 2012). That finding, along with (a) alterations in FMRP distribution in the oocytes of 130R animals, (b) downregulated mTOR signaling in whole ovaries of YAC90R mutants, (c) decreased expression of genes required for ovulation in YAC90 ovaries, and (d) a general elevation in both estradiol and FSH in during adulthood in YAC90R mice all are suggestive of compromised follicle growth and aberrant function.

Whether such aberrant features are also present in ovarian phenotypes that are associated with the *FMR1* gene remains to be seen, but such studies allow hypotheses to be generated and subsequently tested upon human tissue.

It is important to remember that these mouse findings were in the backdrop of overall similar numbers of intact follicles, suggesting that follicles survive, but produce oocytes of poorer “quality” in the presence of the FXPM. Determining whether such a “quality” issue is also at work in women will be important as these women seek out assisted reproductive treatment. Further, our reliance on indirect measures to predict ovarian reserve assumes that all follicles are essentially equal in their production of reserve biomarkers like AMH. If the follicles of women with *FMR1*-associated ovarian aging also contain fewer and fewer healthy granulosa cells, it may be that their follicles produce altered levels of hormones and our prediction(s) as to their ovarian reserve may be less accurate. Continued evaluation of these and future animal models, as well as more direct approaches where follicle numbers are assessed directly in human ovarian cortex (McLaughlin et al., 2011), will allow us to move closer to understanding the biological mechanism(s) behind the ovarian dysfunction seen in relation to the *FMR1* gene.

## Development of functional follicles and oocytes for *in vitro* research

As an alternative to both human research and animal models, patient-specific induced pluripotent stem cells (iPSCs) derived from adult somatic cells and differentiated into GC-like cells represent one novel possible option for generating an abundance of material for research purposes without any invasive procedures. Hayashi et al. showed that functional oocytes could be derived from mouse iPSCs (Hayashi and Sayama, 2009). While this differentiation method relied on *in vivo* co-culture with normal mouse GCs transplanted under the ovarian bursa, the technique showed the feasibility of reconstituting a follicle and generating a functional oocyte from mouse iPSCs.

## Reproductive implications in humans: transmission of FXS

The likelihood that the CGG repeat expands in subsequent generations is important to families of women who carry the premutation and wish to conceive. Thus, we review here the heritability of the *FMR1* gene. Additionally, the heritability of the genotype is important in the context of ovarian reserve if future research confirms an increased risk of infertility among women with a particular repeat length.

The likelihood of a mother with the *FMR1* premutation passing the full mutation to her children increases with the size of her premutation allele. If the mother has approximately 100 repeats of the CGG genetic sequence in the *FMR1* gene, there is a nearly 100% chance that the repeat will expand and her child(ren) will have the full mutation (Nolin et al., 2003, 2011). Risk of expansion to the full mutation is moderated when the *FMR1* CGG repeat is interspersed with one or more AGG's (Nolin et al., 2013). Contractions of the *FMR1* gene have also been reported (Reyniers et al., 1993; Vits et al., 1994; Fisch et al., 1995; Brown et al., 1996; Nolin et al., 1996). Premutation males very rarely pass on a full mutation to their offspring (Ashley-Koch et al., 1998; Zeesman et al., 2004).

The transmission of *FMR1* alleles between mother-offspring pairs (*n* = 238 pairs) is reported to be stable 93.4% of the time in mothers with 45–54 CGG repeats (Cronister et al., 2008). Of the 6.6% that expanded within the intermediate range, the offspring allele size never expanded beyond 60 repeats in 1 generation. The smallest reported repeat size to expand to a full mutation in the subsequent generation was 56; the grandmother of the affected child had a repeat size of 52 (Fernandez-Carvajal et al., 2009). No data are available on the stability of transmission of <45 CGG repeats in females. Thus, while a woman with an intermediate size CGG allele does not need to be concerned with having a child with FXS, her daughter may be at increased risk for *FMR1*-associated ovarian phenotypes, pending the results of future research.

## *FMR1* genetic testing in OB/GYN clinics

The American College of Medical Genetics (ACMG) testing guidelines recommend *FMR1* testing for “women with reproductive or fertility problems associated with elevated FSH levels, especially if there is a family history of POF, FXS, or undiagnosed mental retardation” (Sherman et al., 2005). The National Society of Genetic Counselors and the Genetics Committee of the American College of Obstetrics and Gynecology support this recommendation (ACOG Committee of Genetics, 2010; Finucane et al., 2012), as did participants in a collaborative project between the MIND Institute Fragile X Research and Treatment Center at the University of California at Davis, the National Fragile X Foundation, and the Centers for Disease Control and Prevention (McConkie-Rosell et al., 2007). Some authors have called for further research to explore potential genetic counseling issues for women ascertained in an infertility setting, including the lack of prior experience with individuals with FXS, the impact of unexpected findings on risk perceptions, regret or anger that testing was not considered earlier in the infertility evaluation process, and the shift of focus to include extended family (McConkie-Rosell et al., 2005, 2007; Wittenberger et al., 2007).

There is limited research on emotional reactions to and impact on reproductive decision-making from *FMR1* carrier testing. A study of population screening for *FMR1* premutations (Anido et al., 2005, 2007) found that while women had active coping mechanisms, they also had concerns for the implications of their carrier status for their children or grandchildren, and the results impacted reproductive decisions whether in hindsight or for their own future. Among 20 women with DOR, the emotional reactions to *FMR1* testing were assessed using pre- and post-test questionnaires (Pastore et al., 2008). While participants in this study projected that learning they carried the premutation would have little impact on how they felt about themselves and their self-esteem, most projected that if they did have the premutation they would feel better knowing there was a medical explanation for their infertility. More recently, of 92 women with DOR undergoing *FMR1* testing, 46% thought *FMR1* premutations were “serious” before leaning their test results, however many felt ambivalent or had positive feelings about potentially being a carrier (Pastore et al., 2014c). Women who had never been pregnant thought they were more likely to carry the *FMR1* premutation (*p* = 0.04) and more likely to think the PM is a serious condition (*p* = 0.005) than women who had been pregnant. Thus, clinicians can expect mixed reactions to *FMR1* carrier testing outside of FXS families, with additional differences by her pregnancy history. A qualitative study was conducted on the experience of *FMR1* testing among seven women with DOR and their husbands (Pastore et al., 2014b). Women understood the reproductive implications of carrying the *FMR1* premutation, and hoped for a negative result. Being offered a genetic test caused women to pause and re-think their future reproductive plans. In contrast, the *FMR1* test was viewed as an additional source of information for their husbands as opposed to raising concern regarding potential reproductive ramifications.

## Future directions

Several areas of future research would be very beneficial. Selected suggestions from the authors are noted below:

1. Infertility and *FMR1*: It is unknown if there are potential differences in fertility treatment success by *FMR1* CGG repeat. More broadly, improved risk estimates for a variety of reproductive endpoints would be greatly beneficial for counseling women with a premutation allele. Analogously, risk estimates for women with an intermediate allele would be very informative to patients, genetic counselors, and clinicians.
2. Ovarian hormones and *FMR1*: There is a lack of longitudinal studies of reproductive hormones in *FMR1* premutation carriers. This would be an important step toward providing biomarkers to be used in predicting the severity and timing of ovarian aging in premutation carriers. This may or may not be as informative in women with alleles or other repeat lengths.
3. Genetic counseling in the OB/GYN clinical setting: Genetic counseling issues related to the timing of counseling (pre-test, post-test), the depth of counseling (clinician brief counseling, separate session with genetic counselor, inclusion of family members, etc.), and the content of counseling (e.g., how much information is too much?) are needed.

## Conclusion

This publication reviews the literature on the *FMR1* gene and selected phenotypes involving ovarian dysfunction. The literature is inconsistent on the association between the *FMR1* repeat length and infertility due to low ovarian reserve. Elevated levels of FSH have been found in women who carry the premutation. The literature on the relationship between AMH and the *FMR1* CGG repeat length are contradictory, with variation in the study designs and repeat length under investigation. Genetic counseling issues pertaining to reproduction and the *FMR1* gene are complex, both within and outside of FXS families.

In terms of animal models, two transgeneic mouse lines have been characterized, which allow hypotheses to be tested in ways that are impossible, impractical and/or unethical in humans. Animal models are also under investigation using rats, fruit flies, and nonhuman primates. Research with induced pluripotent stem cells are also under development.

Clearly, further research is needed on the relationship between the *FMR1* gene and reproductive endpoints in women. The inconsistency in the infertility and hormone study findings increases the complexity of genetic counseling for women, and makes it quite difficult for clinicians and patient to interpret the results and anticipate health events. With the increased interest in whole genome sequencing (Howard et al., 2013) and uptake of direct-to-consumer genomic testing (Bloss et al., 2011), the need for meaningful interpretation of genetic results will increase dramatically in the near future. Animal models may provide a means for exploration of the biological mechanism in ways not possible among women, while larger cohort studies and/or more creative study designs may provide the much-needed answers to the impact of the *FMR1* gene on women's reproductive health.

## Funding

Dr. Pastore is partially funded by Eunice K. Shriver National Center for Child Health and Human Development at the National Institutes of Health (grant R01HD068440). The content is solely the responsibility of the authors and does not necessarily represent the official views of the National Institutes of Health.

### Conflict of interest statement

The authors declare that the research was conducted in the absence of any commercial or financial relationships that could be construed as a potential conflict of interest.

## References

[B1] ACOG Committee of Genetics. (2010). Carrier Screening for Fragile X Syndrome. Obstet. Gynecol. 116, 1008–1010 10.1097/AOG.0b013e3181fae884 20859177

[B2] Anido A. Carlson L. M. Sherman S. L. (2007). Attitudes toward fragile X mutation carrier testing from women identified in a general population survey. J. Genet. Couns. 16, 97–104 10.1007/s10897-006-9049-0 17295053

[B3] Anido A. Carlson L. M. Taft L. Sherman S. (2005). Women's attitudes toward testing for fragile X carrier status: a qualitative analysis. J. Genet. Couns. 14, 295–306 10.1007/s10897-005-1159-6 16047092

[B4] Ashley-Koch A. E. Robinson H. Glicksman A. E. Nolin S. L. Schwartz C. E. Brown W. T. (1998). Examination of factors associated with instability of the *FMR1* CGG repeat. Am. J. Hum. Genet. 63, 776–785 10.1086/302018 9718348 PMC1377406

[B5] Barasoain M. Barrenetxea G. Huerta I. Telez M. Carrillo A. Perez C. (2013). Study of *FMR1* gene association with ovarian dysfunction in a sample from the Basque Country. Gene 521, 145–149 10.1016/j.gene.2013.03.032 23537988

[B6] Bloss C. S. Darst B. F. Topol E. J. Schork N. J. (2011). Direct-to-consumer personalized genomic testing. Hum. Mol. Genet. 20, R132–R141 10.1093/hmg/ddr349 21828075 PMC3179383

[B7] Bodega B. Bione S. Dalpra L. Toniolo D. Ornaghi F. Vegetti W. (2006). Influence of intermediate and uninterrupted *FMR1* CGG expansions in premature ovarian failure manifestation. Hum. Reprod. 21, 952–957 10.1093/humrep/dei432 16361284

[B8] Bretherick K. L. Fluker M. R. Robinson W. P. (2005). *FMR1* repeat sizes in the gray zone and high end of the normal range are associated with premature ovarian failure. Hum. Genet. 117, 376–382 10.1007/s00439-005-1326-8 16078053

[B9] Brown W. T. Houck G. E. Jr. Ding X. Zhong N. Nolin S. Glicksman A. (1996). Reverse mutations in the fragile X syndrome. Am. J. Med. Genet. 64, 287–292 8844067 10.1002/(SICI)1096-8628(19960809)64:2<287::AID-AJMG11>3.0.CO;2-B

[B10] Burger H. G. (1994). Diagnostic role of follicle-stimulating hormone (FSH) measurements during the menopausal transition–an analysis of FSH, oestradiol and inhibin. Eur. J. Endocrinol. 130, 38–42 10.1530/eje.0.1300038 8124478

[B11] Burger H. G. (2000). Inhibin and reproductive aging. Exp. Gerontol. 35, 33–39 10.1016/S0531-5565(99)00091-1 10705037

[B12] Burger H. G. Dudley E. Mamers P. Groome N. Robertson D. M. (2000). Early follicular phase serum FSH as a function of age: the roles of inhibin B, inhibin A and estradiol. Climacteric 3, 17–24 10.3109/13697130009167595 11910605

[B13] Bussani C. Papi L. Sestini R. Baldinotti F. Bucciantini S. Bruni V. (2004). Premature ovarian failure and fragile X premutation: a study on 45 women. Eur. J. Obstet. Gynecol. Reprod. Biol. 112, 189–191 10.1016/j.ejogrb.2003.06.003 14746957

[B14] Choe S. A. Kim K. C. Lee J. Y. Kim C. H. Hwang D. Jee B. C. (2013). The relationship between the number of CGG repeats and serum level of anti-Mullerian hormone in women without *FMR1* premutation. Eur. J. Obstet. Gynecol. Reprod. Biol. 169, 275–278 10.1016/j.ejogrb.2013.05.002 23731704

[B15] Coulam C. B. Adamson S. C. Annegars J. F. (1986). Incidence of premature ovarian failure. Obstet. Gynecol. 67, 604–606 3960433

[B16] Cronister A. Teicher J. Rohlfs E. M. Donnenfeld A. Hallam S. (2008). Prevalence and instability of fragile X alleles: implications for offering fragile X prenatal diagnosis. Obstet. Gynecol. 111, 596–601 10.1097/AOG.0b013e318163be0b 18310361

[B17] De Geyter C. M'Rabet N. De Geyter J. Zurcher S. Moffat R. Bosch N. (2013). Similar prevalence of expanded CGG repeat lengths in the fragile X mental retardation I gene among infertile women and among women with proven fertility: a prospective study. Genet. Med. 10.1038/gim.2013.146 24113347

[B18] De Vos M. Devroey P. Fauser B. C. (2010). Primary ovarian insufficiency. Lancet 376, 911–921 10.1016/S0140-6736(10)60355-8 20708256

[B19] Fernandez-Carvajal I. Lopez Posadas B. Pan R. Raske C. Hagerman P. J. Tassone F. (2009). Expansion of an *FMR1* grey-zone allele to a full mutation in two generations. J. Mol. Diagn. 11, 306–310 10.2353/jmoldx.2009.080174 19525339 PMC2710706

[B20] Finucane B. Abrams L. Cronister A. Archibald A. Bennett R. McConkie-Rosell A. (2012). Genetic counseling and testing for *FMR1* gene mutations: practice guidelines of the national society of genetic counselors. J. Genet. Couns. 21, 752–760 10.1007/s10897-012-9524-8 22797890

[B21] Fisch G. S. Snow K. Thibodeau S. N. Chalifaux M. Holden J. J. Nelson D. L. (1995). The fragile X premutation in carriers and its effect on mutation size in offspring. Am. J. Hum. Genet. 56, 1147–1155 7726171 PMC1801463

[B22] Fritz M. A. Speroff L. (2011a). Assisted reproductive technologies, in Clinical Gynecologic Endocrinology and Infertility, 8th Edn. (Philadelphia, PA: Wolters Kluwer Health; Lippincott Williams & Wilkins), 1336–1340

[B23] Fritz M. A. Speroff L. (2011b). Amenorrhea, in Clinical Gynecologic Endocrinology and Infertility, 8th Edn. (Philadelphia, PA: Wolters Kluwer Health; Lippincott Williams & Wilkins), 463–473

[B24] Gleicher N. Weghofer A. Oktay K. Barad D. (2009a). Relevance of triple CGG repeats in the *FMR1* gene to ovarian reserve. Reprod. Biomed. Online 19, 385–390 10.1016/S1472-6483(10)60173-3 19778484

[B25] Gleicher N. Weghofer A. Oktay K. Barad D. H. (2009b). Correlation of triple repeats on the *FMR1* (fragile X) gene to ovarian reserve: a new infertility test? Acta Obstet. Gynecol. Scand. 88, 1024–1030 10.1080/00016340903171058 19642041

[B26] Goldenberg R. L. Grodin J. M. Rodbard D. Ross G. T. (1973). Gonadotropins in women with amenorrhea. The use of plasma follicle-stimulating hormone to differentiate women with and without ovarian follicles. Am. J. Obstet. Gynecol. 116, 1003–1012 4736951 10.1016/s0002-9378(16)33850-9

[B27] Hansen K. R. Hodnett G. M. Knowlton N. Craig L. B. (2011). Correlation of ovarian reserve tests with histologically determined primordial follicle number. Fertil. Steril. 95, 170–175 10.1016/j.fertnstert.2010.04.006 20522327

[B28] Hayashi H. Sayama M. (2009). Emotional processes during pregnancy among women successfully conceived via assisted reproductive technology [Japanese]. J. Japan Acad. Midwifery 23, 83–92 10.3418/jjam.23.83

[B29] Hoffman G. E. Le W. W. Entezam A. Otsuka N. Tong Z. B. Nelson L. (2012). Ovarian abnormalities in a mouse model of fragile X primary ovarian insufficiency. J. Histochem. Cytochem. 60, 439–456 10.1369/0022155412441002 22470123 PMC3393073

[B30] Howard H. C. Swinnen E. Douw K. Vondeling H. Cassiman J. J. Cambon-Thomsen A. (2013). The ethical introduction of genome-based information and technologies into public health. Public Health Genomics 16, 100–109 10.1159/000346474 23428828

[B31] Hundscheid R. D. Braat D. D. Kiemeney L. A. Smits A. P. Thomas C. M. (2001). Increased serum FSH in female fragile X premutation carriers with either regular menstrual cycles or on oral contraceptives. Hum. Reprod. 16, 457–462 10.1093/humrep/16.3.457 11228211

[B32] Jameson J. DeGroot L. (2010). Amenorrhea, anovulation, and dysfunctional uterine bleeding, in Endocrinology: Adult and Pediatric, 6th Edn., Vol. 2, eds DeGroot L. J. Jameson J. L. De Kretser D. M. Grossman A. Marshall J. C. Melmed S. Potts J. T. Weir G. C. (Philadelphia, PA: Saunders/Elsevier), 2347–2350

[B33] Karimov C. B. Moragianni V. A. Cronister A. Srouji S. Petrozza J. Racowsky C. (2011). Increased frequency of occult fragile X-associated primary ovarian insufficiency in infertile women with evidence of impaired ovarian function. Hum. Reprod. 26, 2077–2083 10.1093/humrep/der168 21646280

[B34] Klein N. A. Battaglia D. E. Woodruff T. K. Padmanabhan V. Giudice L. C. Bremner W. J. (2000). Ovarian follicular concentrations of activin, follistatin, inhibin, insulin-like growth factor I (IGF-I), IGF-II, IGF-binding protein-2 (IGFBP-2), IGFBP-3, and vascular endothelial growth factor in spontaneous menstrual cycles of normal women of advanced reproductive age. J. Clin. Endocrinol. Metab. 85, 4520–4525 10.1210/jcem.85.12.7056 11134102

[B35] Kline J. Kinney A. Kelly A. Reuss M. L. Levin B. (2005). Predictors of antral follicle count during the reproductive years. Hum. Reprod. 20, 2179–2189 10.1093/humrep/dei048 15860491

[B36] Kline J. K. Kinney A. M. Levin B. Brown S. A. Hadd A. G. Warburton D. (2014). Intermediate CGG repeat length at the *FMR1* locus is not associated with hormonal indicators of ovarian age. Menopause 21, 740–748 10.1097/GME.0000000000000139 24423935 PMC4065625

[B37] Kronquist K. E. Sherman S. L. Spector E. B. (2008). Clinical significance of tri-nucleotide repeats in Fragile X testing: a clarification of American College of Medical Genetics guidelines. Genet. Med. 10, 845–847 10.1097/GIM.0b013e31818c2606 18941415 PMC3111547

[B38] Lu C. Lin L. Tan H. Wu H. Sherman S. L. Gao F. (2012). Fragile X premutation RNA is sufficient to cause primary ovarian insufficiency in mice. Hum. Mol. Genet. 21, 5039–5047 10.1093/hmg/dds348 22914733 PMC3490511

[B39] Maddalena A. Richards C. S. McGinniss M. J. Brothman A. Desnick R. J. Grier R. E. (2001). Technical standards and guidelines for fragile X: the first of a series of disease-specific supplements to the Standards and Guidelines for Clinical Genetics Laboratories of the American College of Medical Genetics. Quality Assurance Subcommittee of the Laboratory Practice Committee. Genet. Med. 3, 200–205 10.1097/00125817-200105000-00010 11388762 PMC3110344

[B40] Marozzi A. Vegetti W. Manfredini E. Tibiletti M. G. Testa G. Crosignani P. G. (2000). Association between idiopathic premature ovarian failure and fragile X premutation. Hum. Reprod. 15, 197–202 10.1093/humrep/15.1.197 10611212

[B41] McConkie-Rosell A. Abrams L. Finucane B. Cronister A. Gane L. W. Coffey S. M. (2007). Recommendations from Multi-disciplinary Focus Groups on Cascade Testing and Genetic Counseling for Fragile X-associated Disorders. J. Genetic. Couns. 16, 593–606 10.1007/s10897-007-9099-y 17497108

[B42] McConkie-Rosell A. Finucane B. Cronister A. Abrams L. Bennett R. L. Pettersen B. J. (2005). Genetic counseling for fragile x syndrome: updated recommendations of the national society of genetic counselors. J. Genetic. Couns. 14, 249–270 10.1007/s10897-005-4802-x 16047089

[B43] McLaughlin M. Patrizio P. Kayisli U. Luk J. Thomson T. C. Anderson R. A. (2011). mTOR kinase inhibition results in oocyte loss characterized by empty follicles in human ovarian cortical strips cultured *in vitro*. Fertil. Steril. 96, 1154.e1–1159.e1 10.1016/j.fertnstert.2011.08.040 22036052

[B44] Monaghan K. G. Lyon E. Spector E. B. (2013). ACMG Standards and Guidelines for fragile X testing: a revision to the disease-specific supplements to the Standards and Guidelines for Clinical Genetics Laboratories of the American College of Medical Genetics and Genomics. Genet. Med. 15, 575–586 10.1038/gim.2013.61 23765048

[B45] Murray A. Ennis S. MacSwiney F. Webb J. Morton N. E. (2000). Reproductive and menstrual history of females with fragile X expansions. Eur. J. Hum. Genet. 8, 247–252 10.1038/sj.ejhg.5200451 10854106 PMC3725540

[B46] Murray A. Schoemaker M. J. Bennett C. E. Ennis S. Macpherson J. N. Jones M. (2014). Population-based estimates of the prevalence of *FMR1* expansion mutations in women with early menopause and primary ovarian insufficiency. Genet. Med. 16, 19–24 10.1038/gim.2013.64 23703681 PMC3914024

[B47] Murray A. Webb J. MacSwiney F. Shipley E. L. Morton N. E. Conway G. S. (1999). Serum concentrations of follicle stimulating hormone may predict premature ovarian failure in FRAXA premutation women. Hum. Reprod. 14, 1217–1218 10.1093/humrep/14.5.1217 10325264 PMC3728645

[B48] Nelson L. M. Anasti J. N. Kimzey L. M. Defensor R. A. Lipetz K. J. White B. J. (1994). Development of luteinized graafian follicles in patients with karyotypically normal spontaneous premature ovarian failure. J. Clin. Endocrinol. Metab. 79, 1470–1475 7962345 10.1210/jcem.79.5.7962345

[B49] Nelson S. M. La Marca A. (2011). The journey from the old to the new AMH assay: how to avoid getting lost in the values. Reprod. Biomed. Online 23, 411–420 10.1016/j.rbmo.2011.06.011 21872528

[B50] Nolin S. L. Brown W. T. Glicksman A. Houck G. E. Jr. Gargano A. D. Sullivan A. (2003). Expansion of the fragile X CGG repeat in females with premutation or intermediate alleles. Am. J. Hum. Genet. 72, 454–464 10.1086/367713 12529854 PMC379237

[B51] Nolin S. L. Glicksman A. Ding X. Ersalesi N. Brown W. T. Sherman S. L. (2011). Fragile X analysis of 1112 prenatal samples from 1991 to 2010. Prenat. Diagn. 31, 925–931 10.1002/pd.2815 21717484

[B52] Nolin S. L. Lewis F. A. 3rd. Ye L. L. Houck G. E. Jr. Glicksman A. E. Limprasert P. (1996). Familial transmission of the *FMR1* CGG repeat. Am. J. Hum. Genet. 59, 1252–1261 8940270 PMC1914886

[B53] Nolin S. L. Sah S. Glicksman A. Sherman S. L. Allen E. Berry-Kravis E. (2013). Fragile X AGG analysis provides new risk predictions for 45-69 repeat alleles. Am. J. Med. Genet. A 161, 771–778 10.1002/ajmg.a.35833 23444167 PMC4396070

[B54] Oostra B. A. Willemsen R. (2003). A fragile balance: *FMR1* expression levels. Hum. Mol. Genet. 12, R249–R257 10.1093/hmg/ddg298 12952862

[B55] Otsuka S. Sakamoto Y. Siomi H. Itakura M. Yamamoto K. Matumoto H. (2010). Fragile X carrier screening and *FMR1* allele distribution in the Japanese population. Brain Dev. 32, 110–114 10.1016/j.braindev.2008.12.015 19211207

[B57a] Pastore L. M. Antero M. F. Ventura K. A. Penberthy J. K. Thomas S. A. Karns L. B. (2014c). Attitudes towards potentially carrying the FMR1 premutation: before vs after testing of non-carrier females with occult primary ovarian insufficiency (POI). J. Genet. Couns. [Epub ahead of print] 10.1007/s10897-014-9717-4 PMC5225992 24788194

[B56] Pastore L. M. Karns L. B. Ventura K. Clark M. L. Steeves R. H. Callanan N. P. (2014b). Longitudinal interviews of couples diagnosed with diminished ovarian reserve undergoing Fragile X Premutation testing. J. Genet. Couns. 23, 97–107 10.1007/s10897-013-9616-0 23764957 PMC3800506

[B57] Pastore L. M. McMurry T. L. Williams C. D. Baker V. B. Young S. L. (2014a). AMH in women with diminished ovarian reserve: potential differences by *FMR1* CGG repeat level. J. Assist. Repro. Genet. 10.1007/s10897-014-9717-4 24938362 PMC4171418

[B58] Pastore L. M. Morris W. L. Karns L. B. Pastore L. M. Morris W. L. Karns L. B. (2008). Emotional reaction to fragile X premutation carrier tests among infertile women. J. Genet. Couns. 17, 84–91 10.1007/s10897-007-9129-9 17968639

[B59] Pastore L. M. Young S. L. Baker V. L. Karns L. B. Williams C. D. Silverman L. M. (2012). Elevated prevalence of 35-44 *FMR1* trinucleotide repeats in women with diminished ovarian reserve. Reprod. Sci. 19, 1226–1231 10.1177/1933719112446074 22581803 PMC4051402

[B60] Rebar R. W. Connolly H. V. (1990). Clinical features of young women with hypergonadotropic amenorrhea. Fertil. Steril. 53, 804–810 2110072

[B61] Reyniers E. Vits L. De Boulle K. Van Roy B. Van Velzen D. de Graaff E. (1993). The full mutation in the *FMR-1* gene of male fragile X patients is absent in their sperm. Nat. Genet. 4, 143–146 10.1038/ng0693-143 8348152

[B62] Rohr J. Allen E. G. Charen K. Giles J. He W. Dominguez C. (2008). Anti-Mullerian hormone indicates early ovarian decline in fragile X mental retardation (*FMR1*) premutation carriers: a preliminary study. Hum. Reprod. 23, 1220–1225 10.1093/humrep/den050 18310677

[B63] Seltzer M. M. Abbeduto L. Greenberg J. S. Almeida D. Hong J. Witt W. (2009). Biomarkers in the study of families of individuals with developmental disabilities. Int. Rev. Res. Ment. Retard. 37, 213–249 10.1016/S0074-7750(09)37007-X 20414357 PMC2856101

[B65] Sherman S. Pletcher B. A. Driscoll D. A. (2005). Fragile X syndrome: diagnostic and carrier testing. Genet. Med. 7, 584–587 10.1097/01.GIM.0000182468.22666.dd 16247297 PMC3110946

[B66] Spath M. A. Feuth T. B. Allen E. G. Smits A. P. Yntema H. G. van Kessel A. G. (2011). Intra-individual stability over time of standardized anti-Mullerian hormone in *FMR1* premutation carriers. Hum. Reprod. 26, 2185–2191 10.1093/humrep/der146 21576079 PMC3137386

[B67] Streuli I. Fraisse T. Ibecheole V. Moix I. Morris M. A. de Ziegler D. (2009). Intermediate and premutation *FMR1* alleles in women with occult primary ovarian insufficiency. Fertil. Steril. 92, 464–470 10.1016/j.fertnstert.2008.07.007 18973899

[B68] Sullivan A. K. Marcus M. Epstein M. P. Allen E. G. Anido A. E. Paquin J. J. (2005). Association of *FMR1* repeat size with ovarian dysfunction. Hum. Reprod. 20, 402–412 10.1093/humrep/deh635 15608041

[B69] van Rooij I. Broekmans F. J. Scheffer G. J. Looman C. W. Habbema J. D. de Jong F. H. (2005). Serum antimullerian hormone levels best reflect the reproductive decline with age in normal women with proven fertility: a longitudinal study. Fertil. Steril. 83, 979–987 10.1016/j.fertnstert.2004.11.029 15820810

[B70] Visser J. A. Themmen A. P. (2005). Anti-Mullerian hormone and folliculogenesis. Mol. Cell. Endocrinol. 234, 81–86 10.1016/j.mce.2004.09.008 15836956

[B71] Vits L. De Boulle K. Reyniers E. Handig I. Darby J. K. Oostra B. (1994). Apparent regression of the CGG repeat in *FMR1* to an allele of normal size. Hum. Genet. 94, 523–526 10.1007/BF00211019 7959688

[B72] Weenen C. Laven J. S. Von Bergh A. R. Cranfield M. Groome N. P. Visser J. A. (2004). Anti-Mullerian hormone expression pattern in the human ovary: potential implications for initial and cyclic follicle recruitment. Mol. Hum. Reprod. 10, 77–83 10.1093/molehr/gah015 14742691

[B73] Welt C. K. (2008). Primary ovarian insufficiency: a more accurate term for premature ovarian failure. Clin. Endocrinol. (Oxf.) 68, 499–509 10.1111/j.1365-2265.2007.03073.x 17970776

[B74] Welt C. K. Smith P. C. Taylor A. E. (2004). Evidence of early ovarian aging in Fragile X Premutation carriers. J. Clin. Endocrinol. Metab. 89, 4569–4574 10.1210/jc.2004-0347 15356064

[B75] Wittenberger M. D. Hagerman R. J. Sherman S. L. McConkie-Rosell A. Welt C. K. Rebar R. W. (2007). The *FMR1* premutation and reproduction. Fertil. Steril. 87, 456–465 10.1016/j.fertnstert.2006.09.004 17074338

[B76] Yu J. Yaba A. Kasiman C. Thomson T. Johnson J. (2011). mTOR controls ovarian follicle growth by regulating granulosa cell proliferation. PLoS ONE 6:e21415 10.1371/journal.pone.0021415 21750711 PMC3130037

[B77] Zeesman S. Zwaigenbaum L. Whelan D. T. Hagerman R. J. Tassone F. Taylor S. A. M. (2004). Paternal transmission of fragile X syndrome. Am. J. Med. Genet. A 129A, 184–189 10.1002/ajmg.a.30191 15316964

